# Targeting myocardial inflammation: investigating the therapeutic potential of atrial natriuretic peptide in atrial fibrosis

**DOI:** 10.1007/s11033-024-09393-w

**Published:** 2024-04-15

**Authors:** Nana Zhu, Tianlun Li, Yili Bai, Jiao Sun, Jianping Guo, Hongtao Yuan, Zhaoliang Shan

**Affiliations:** 1 Graduate School, Medical School of Chinese PLA, Beijing, China; 2 Southern Medical Branch, Chinese PLA General Hospital, Beijing, China; 3 Department of Cardiovascular Medicine, the Sixth Medical Centre, Chinese PLA General Hospital, Beijing, China

**Keywords:** Atrial fibrosis, Atrial fibroblasts, ANP, Inflammation, Tenascin-C

## Abstract

**Background:**

Atrial Fibrillation (AF), a prevalent arrhythmic condition, is intricately associated with atrial fibrosis, a major pathological contributor. Central to the development of atrial fibrosis is myocardial inflammation. This study focuses on Atrial Natriuretic Peptide (ANP) and its role in mitigating atrial fibrosis, aiming to elucidate the specific mechanisms by which ANP exerts its effects, with an emphasis on fibroblast dynamics.

**Methods and results:**

The study involved forty Sprague-Dawley rats, divided into four groups: control, Angiotensin II (Ang II), Ang II + ANP, and ANP only. The administration of 1 µg/kg/min Ang II was given to Ang II and Ang II + ANP groups, while both Ang II + ANP and ANP groups received 0.1 µg/kg/min ANP intravenously for a duration of 14 days. Cardiac fibroblasts were used for in vitro validation of the proposed mechanisms. The study observed that rats in the Ang II and Ang II + ANP groups showed an increase in blood pressure and a decrease in body weight, more pronounced in the Ang II group. Diastolic dysfunction, a characteristic of the Ang II group, was alleviated by ANP. Additionally, ANP significantly reduced Ang II-induced atrial fibrosis, myofibroblast proliferation, collagen overexpression, macrophage infiltration, and the elevated expression of Interleukin 6 (IL-6) and Tenascin-C (TN-C). Transcriptomic sequencing indicated enhanced PI3K/Akt signaling in the Ang II group. Furthermore, in vitro studies showed that ANP, along with the PI3K inhibitor LY294002, effectively reduced PI3K/Akt pathway activation and the expression of TN-C, collagen-I, and collagen-III, which were induced by Ang II.

**Conclusions:**

The study demonstrates ANP’s potential in inhibiting myocardial inflammation and reducing atrial fibrosis. Notably, ANP’s effect in countering atrial fibrosis seems to be mediated through the suppression of the Ang II-induced PI3K/Akt-Tenascin-C signaling pathway. These insights enhance our understanding of AF pathogenesis and position ANP as a potential therapeutic agent for treating atrial fibrosis.

**Supplementary Information:**

The online version contains supplementary material available at 10.1007/s11033-024-09393-w.

## Background

Atrial fibrillation, one of the most common supraventricular arrhythmias, poses significant clinical challenges, being closely linked to increased mortality and associated with the onset of heart failure, stroke, and cognitive impairment [1, 2]. The global incidence of AF is escalating, a trend attributable to the aging population and a rise in cardiovascular risk factors [3]. Currently, the global prevalence of AF is approximately 60 million cases and contributes to > 8 million disability-adjusted life years [4], emphasizing the urgency of developing effective treatments. Pulmonary vein isolation stands as a leading therapeutic approach to restore sinus rhythm in AF patients. However, the efficacy of this treatment varies, ranging from 50 to 80% [5–7], underscoring the need for more effective and consistent treatment strategies, particularly in managing AF recurrence following radiofrequency ablation.

The pathogenesis of AF is complex, involving multiple factors such as atrial remodeling, inflammation, oxidative stress, genetics, and autonomic nerve remodeling. Among these, atrial remodeling, which encompasses both electrical and structural changes, is a pivotal pathological contributor to AF [8]. A key component of this structural remodeling is atrial fibrosis, characterized by aberrant fibroblast activation, proliferation, differentiation, and the excessive synthesis and irregular deposition of extracellular matrix proteins. Atrial fibrosis is known to induce local conduction block and foster reentry, thereby contributing significantly to the onset and persistence of AF. Furthermore, studies have shown a direct correlation between the degree of atrial fibrosis and the recurrence of AF post-radiofrequency ablation, where even a 1% increase in fibrosis can lead to a 6% rise in recurrence risk [9].

The intricate relationship between chronic inflammation and atrial fibrosis is well-documented, with several factors associated with chronic inflammation identified as primary contributors to atrial fibrosis. The renin-angiotensin-aldosterone system (RAAS), in particular, plays a central role in this process, with Ang II being a key mediator within the RAAS [10–12]. Traditionally, Ang II is believed to activate the TGF-β/Smad-2/3 pathway, leading to fibroblast differentiation, proliferation, and secretion, thereby promoting atrial fibrosis development. Recent research has increasingly highlighted the critical role of RAAS-mediated inflammation in myocardial fibrosis. In this context, TN-C, an extracellular matrix glycoprotein that exhibits minimal expression in normal adult hearts but is substantially upregulated during tissue injury and inflammation, emerges as a potent marker for inflammatory diseases and plays a crucial role in the processes of fibrosis and inflammation [13–15].

ANP, synthesized and secreted by atrial cells, particularly from the atrial appendage, emerges as a molecule of interest. Its secretion is stimulated by the stretching of the left atrium and is influenced by angiotensin and endothelin. Elevated atrial volume or pressure load leads to a significant increase in ANP secretion due to atrial stretching [16] . ANP operates through binding to its receptors—NP receptor types A, B, and C—inducing various physiological responses. Known for its diuretic, natriuretic, antihypertensive, and anti-cardiac hypertrophy effects, ANP has also been correlated with improved renal function post-cardiac surgery, offering renal protection, reducing pulmonary artery pressure, and alleviating cardiac reperfusion injury [17]. Recent investigations have expanded ANP’s profile, suggesting its role in immune modulation and anti-inflammatory functions, indicating its potential as a hormone-like anti-inflammatory cytokine. Studies have shown that administering ANP post-myocardial infarction can reduce inflammation in the affected myocardial region, decrease fibrosis, and consequently reduce the infarcted area’s extent [18]. Therefore, ANP may be a potential effective therapeutic approach to inflammation and fibrosis in AF.

This study aims to investigate whether ANP can mitigate Ang II-induced atrial inflammation and fibrosis in a rat model and to explore the associated molecular mechanisms, particularly focusing on the inflammatory factor TN-C. The research endeavors to provide novel evidence supporting ANP’s role as an anti-inflammatory factor in the treatment of atrial fibrosis and to lay a theoretical foundation for the clinical development of drugs targeting the prevention and treatment of atrial fibrosis.

## Materials and methods

### Animals

The animals used in this study were bred and maintained at the Laboratory of Animal Experiments, Chinese PLA General Hospital. They were fed a standard rat-chow diet with ad libitum access to tap water. All experimental procedures adhered to the guidelines of the US National Institutes of Health Guide for the Care and Use of Laboratory Animals (NIH Publication No. 85 − 23, 1996). The study protocols were also ethically reviewed and approved by the Animal Care and Use Committee of the Chinese PLA General Hospital.

### Animal models

Under standard experimental conditions (20–25 °C, 50-60% relative humidity, and a 12-hour light/12-hour dark cycle), 8-week-old Sprague-Dawley (SD) rats, each weighing approximately 250 g ± 10 g, were used. These rats were randomly divided into four groups: control, Ang II, Ang II + ANP, and ANP group. A midline incision in the lumbar region was performed for the insertion of osmotic mini-pumps (model 2002; ALZET, Palo Alto, CA, USA; average filling volume 0.234 ml). In the control group, saline was administered via these pumps at a rate of 1 µg/kg/min over a 14-day period. For the Ang II and Ang II + ANP groups, osmotic mini-pumps containing Ang II (MedChemExpress, Monmouth, NJ) were used to infuse at a rate of 1 µg/kg/min for the same duration. The Ang II + ANP and ANP groups received mini-pumps filled with carperitide, a recombinant α-human ANP (MedChemExpress, State of New Jersey, USA), dissolved in distilled water and released at a rate of 0.1 µg/kg/min over 14 days, connected to the right or left jugular vein using a small polyethylene catheter [19–20]. Rat weights were systematically documented on days 0, 7, and 14, and systolic blood pressure was measured on these days using a tail-cuff method without anesthesia. On day 14, the rats were deeply anesthetized with chloral hydrate and euthanized for heart dissection and subsequent experimental procedures.

### Echocardiography

Transthoracic echocardiography was performed on the rats from each experimental group on day 14. To ensure accuracy and consistency of data, all experimental rats were subjected to echocardiographic examination by the same experienced physician specializing in ultrasonography. For anesthesia, rats were subjected to 2.5% isoflurane using nose cone administration, maintained at 1.5% isoflurane throughout the procedure. The echocardiographic assessments utilized an MS250 probe with the Vevo 2100 software package (FUJIFILM VisualSonics, USA). The procedure involved direct measurements of end-diastolic left ventricular dimension (LVDd) and end-systolic left ventricular dimension (LVDs), which are crucial indicators of heart function. The left ventricular ejection fraction (EF) was calculated as a percentage, derived from the LVDd and LVDs measurements. This calculation is essential for assessing the heart’s pumping efficiency.In addition to these measurements, diastolic function was evaluated by measuring the peak velocity of mitral early inflow (E) and calculating its ratio to the peak velocity of atrial contraction-related flow (E/A). These measurements provide valuable insights into the diastolic performance of the heart, an important aspect of cardiac function in the context of atrial fibrillation and fibrosis.

### Histology and immunohistochemistry

Post-euthanasia, the hearts were quickly excised from the rats, and atrial tissues were dissected and fixed in 4% paraformaldehyde, preparing them for pathological examination. Atrial fibrosis was quantified using Masson’s trichrome staining, a method recognized for its precision in identifying and measuring collagen deposition. Digital images of the stained tissue sections were processed using Image J, and each atrial section was photographed at 200x magnification. From these, ten fields of view were randomly selected for detailed analysis, allowing for the quantification of the fibrotic area as a percentage of the total atrial tissue area. Furthermore, considering the atrium as a holistic entity without distinguishing between the left and right atrial, this comprehensive approach was primarily adopted to assess the cumulative impact of Ang-II on atrial fibrosis, rather than exploring inter-atrial differences. For Immunohistochemistry (IHC), the expression levels of alpha-smooth muscle actin (α-SMA) and macrophage presence were assessed. A rabbit monoclonal antibody specific for α-SMA (1:200 dilution, Abcam, Cambridge, MA) and another targeting the macrophage marker CD68 (1:100 dilution, Abcam, Cambridge, MA) were used. As a control, an irrelevant isotype goat IgG was employed (1:200 dilution, ZSGB-BIO, Beijing, China). Digital images of the stained sections were captured at 200x magnification, covering over 10 random fields per section. The quantification of positive areas was carried out in a double-blind manner to ensure unbiased results.

### RNA extraction and real-time PCR analysis

To analyze the mRNA expression levels of collagen type I, collagen type III, TN-C, IL-6, and glyceraldehyde-3-phosphate dehydrogenase (GAPDH), real-time reverse transcription-polymerase chain reaction (real-time RT-PCR) was employed. Following total RNA isolation from the atrial tissues, quantitative PCR analysis was performed using the LightCycler system (LightCycler FastStart DNA Master PLUS SYBR Green I; Roche Diagnostics; Mannheim, Germany). A melting curve analysis, as per the manufacturer’s instructions, was conducted to ensure the specificity of the amplification. The normalization of mRNA expression for each target gene was relative to GAPDH expression levels. The sequences of the forward and reverse primers used in this analysis are detailed in Table 1.

**Table 1:** Oligonucleotide primers used for real-time RT-PCR

Gene	Primers
Collagen type I	
Forward	5’-GAGCATTTGCCTCGGTGTCTAC-3’
Reverse	5’-GCAGGGATGGGAAAGTCAAA-3’
Collagen type III	
Forward	5’-GGACAAATAGAGAGTCTTATCAGCCC-3’
Reverse	5’-TTATAGCATCCATCTTGCAGCCT-3’
TGF-β	
Forward	5’-CATTTGGAGCCTGGACACACA-3’
Reverse	5’-GCTTGCGACCCACGTAGTAGAC-3’
TN-C	
Forward	5’-GGGTCCTCAAGAAAGTCA-3’
Reverse	5’-TCCTCTGGCAAGGTCAAG-3’
IL-6	
Forward	5’-GGCCCTTGCTTTCTCTTCG-3’
Reverse	5’-ATAATAAAGTTTTGATTATGT-3’
GAPDH	
Forward	5’-CGTATCGGACGCCTGGTT-3’
Reverse	5’-AGGTCAATGAAGGGGTCGTT-3’

### Eukaryotic transcriptome sequencing

For transcriptome analysis, RNA was extracted from rat atrial tissue samples. The quality of the extracted RNA was assessed using the RNA Nano 6000 Assay Kit on an Agilent Bioanalyzer 2100 system (Agilent Technologies, CA, USA). For RNA sample preparations, a standardized input of 1 µg of RNA per sample was used. Sequencing libraries were then constructed using the NR604-VAHTS® Fast RNA-seq Library Prep Kit for Illumina (Vazyme, China), adhering to the manufacturer’s protocols. The initial sequencing reads were subjected to rigorous quality control to filter out low-quality reads, adapter contamination, and reads containing more than 10% unknown bases.Gene expression levels were quantified using the Fragments Per Kilobase of transcript per Million mapped reads (FPKM) metric. Genes were identified as significantly differentially expressed if they demonstrated an absolute Log2 fold change ≥ 0 and a false discovery rate (FDR) ≤ 0.05. For data visualization and analysis, a heatmap was generated using the R package pheatmap. Molecular interaction network analysis included protein–protein interaction (PPI) network assessment through STRING (https://www.string-db.org/) and Ingenuity Pathway Analysis (IPA, Qiagen Redwood City Inc., CA, USA). Gene function and pathway enrichment analyses were performed using the Database for Annotation, Visualization, and Integrated Discovery (DAVID) (https://david.abcc.ncifcrf.gov/), as well as the Cytoscape plug-ins ClueGO and CluePedia. The significance levels for terms and pathways were determined based on a false discovery rate (FDR) threshold of ≤ 0.05.

### Cell culture

Cardiac fibroblasts were isolated from the atria of rats and cultured in Dulbecco’s Modified Eagle Medium (DMEM/F12) supplemented with 10% fetal bovine serum (MedChemExpress, Monmouth, NJ), according to established protocols [21]. For the experiments, cells from secondary cultures were used. When the fibroblasts in a 10 cm culture dish reached 90% confluency, the existing medium was discarded. The cells were then subjected to serum starvation for 24 h using fresh DMEM without fetal bovine serum, synchronizing the cells. After this period, the cells were treated with different concentrations of Ang II (ranging from 1 × 10^-9 to 1 × 10^-5 mol/l) for 24 h to identify the optimal concentration affecting fibroblast cells. Additionally, LY294002 (1 × 10^-5 mol/l, MedChemExpress, Monmouth, NJ), a PI3K antagonist, was used to explore the signaling pathways activated by Ang II. To investigate the inhibitory effects of ANP and LY294002 on Ang II-induced TN-C and collagen expression, cells were pre-treated with ANP (1 × 10^-4 mol/l) or LY294002 (1 × 10^-5 mmol/L) for 1 h before Ang II stimulation (1 × 10^-6 mol/l) for 24 h. Western blotting was utilized to measure the expression levels of collagen type I, collagen type III, TN-C, GAPDH, and proteins involved in the signaling pathway.

### Western blot analysis

Total protein from fresh tissues or cells was lysed using a buffer containing RIPA buffer, 1% protein phosphatase inhibitor (Solarbio, Beijing, China), and 1% phenylmethanesulfonyl fluoride (PMSF, Solarbio, Beijing, China). Protein concentrations were quantified using a BCA Protein assay kit (Solarbio, Beijing, China). Proteins (20 micrograms) were separated on a 4–12% SDS-PAGE gel and transferred to polyvinylidene difluoride membranes (Merck Millipore) at a current of 300 mA for 95 min. After transfer, the membranes were blocked and incubated with primary antibodies overnight at 4 °C. The primary antibodies used included anti-GAPDH (1:5000, Solarbio), anti-COL-I (1:2000, Abcam), anti-COL-III (1:2000, Abcam), anti- Transforming growth factor -β(TGF-β)(1:3000,Abcam),anti-TN-C (1:2000, Huaxingbio, Beijing), and anti-IL-6 (1:2000, Abcam). This was followed by a 1-hour room temperature incubation with IR Dye-conjugated secondary antibodies (1:5000, ZSGB-BIO, Beijing). The membranes were then imaged and quantified using an Odyssey infrared imaging system (LI-COR Biosciences, Lincoln, NE). To ensure robustness and reliability of data, the entire experimental procedure was repeated three times.

### Statistical analysis

The analysis of sequencing data was conducted using a series of bioinformatics tools. Initially, raw sequencing data were filtered to obtain high-quality sequences (Clean Data), which were then aligned with the reference genome of the respective species. This alignment facilitated the calculation of gene expression levels for each gene. Subsequent analyses included differential expression analysis, enrichment analysis, and clustering analysis of the samples. The data in this study are presented as mean ± standard error of the mean (SEM). Differences between groups were evaluated using Student’s t-test or analysis of variance (ANOVA). When ANOVA indicated significant differences, a Newman-Keuls Multiple Comparison Test was employed for post-hoc analysis. These statistical tests were conducted using GraphPad Prism version 9.0 software. A p-value of less than 0.05 (*P* < 0.05) was considered statistically significant in all analyses. This threshold was used to determine the presence of statistically significant differences between the experimental groups.

## Results

### Effects of ANP on blood pressure and weight changes induced by Ang II

**Fig. 1:**
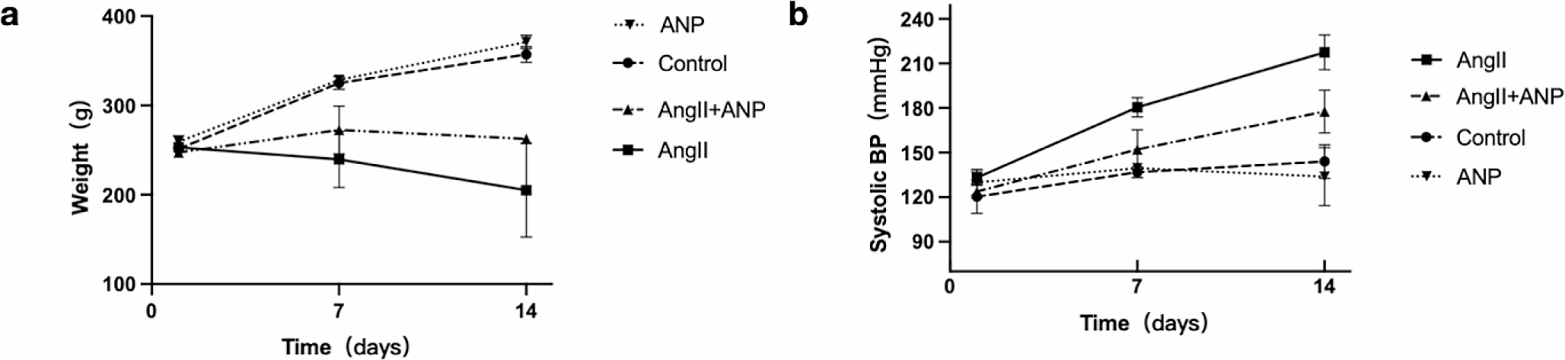
The difference of systolic blood pressure (BP) and weight. (**a**) The time course of systolic blood pressure (BP) (**b**) The time course of weight. Control: control rats, Ang II: angiotensin II-treated rats, Ang II + ANP: Ang II plus atrial natriuretic peptide-treated rats, ANP: atrial natriuretic peptide-treated rats

The study observed significant effects of Ang II and its combination with ANP on systolic blood pressure (SBP), body weight, and heart weight/body weight ratio in rats. Compared to control rats (SBP: 144.0 ± 11.29 mmHg), rats treated with Ang II showed a substantial increase in SBP (217.5 ± 11.78 mmHg), which was notably improved in the Ang II + ANP group (177.6 ± 14.34 mmHg; *P* < 0.01). Similarly, body weight was significantly reduced in the Ang II group (205.0 ± 52.60 g) compared to controls (357.0 ± 8.89 g), with a less pronounced reduction in the Ang II + ANP group (262.7 ± 2.89 g; *P* < 0.05). Interestingly, the increase in blood pressure and reduction in body weight were more severe in the Ang II-only group. In contrast, no significant differences were observed in the group treated with ANP alone, in terms of systolic blood pressure (133.8 ± 19.59 mmHg) and body weight (371.0 ± 7.56 g) compared to controls. These findings are illustrated in Fig. 1 and detailed in Table 3.

### ANP’s protective effect on cardiac function

The study revealed significant findings regarding ANP’s role in cardiac function, particularly in terms of the E/A ratio, a key indicator of diastolic function. Compared to control rats, which had an E/A ratio of 1.51 ± 0.29, rats treated with Ang II demonstrated a marked decrease in this ratio (0.68 ± 0.18*P* < 0.01). Notably, the infusion of ANP in the Ang II + ANP group led to a correction of the E/A ratio to 1.24 ± 0.07 (*P* < 0.05), indicating an improvement in cardiac diastolic function in these rats. In terms of ejection fraction (EF), ventricular wall thickness, and other cardiac parameters, there were no significant differences observed among the various rat groups. To provide a comprehensive overview, Table 2 summarizes the systolic blood pressure, body weight, heart weight/body weight ratio, and echocardiographic parameters for each group of rats on day 14.

**Table 2:** Systolic blood pressure, body weight, heart weight/body weight ratio, and echocardiographic parameters on day 14

	Control	Ang II	Ang II + ANP	ANP
Systolic blood pressure (mmHg)	144.0 ± 11.29	217.5 ± 11.78**	177.6 ± 14.34*^△^	133.8 ± 19.59
Body weight (g)	357.0 ± 8.89	205.0 ± 52.60**	262.7 ± 2.89*^△^	371.0 ± 7.56
LVPW; d (mm)	2.24 ± 0.23	2.16 ± 0.50	2.34 ± 0.34	1.71 ± 0.15
LVPW; s (mm)	3.38 ± 0.53	3.98 ± 0.36	3.56 ± 0.13	2.86 ± 0.46
LVD; d (mm)	6.25 ± 0.53	5.87 ± 0.17	6.12 ± 1.54	7.38 ± 0.92
LVD; s (mm)	2.99 ± 0.65	1.88 ± 0.84	2.76 ± 1.04	3.85 ± 0.867
EF (%)	86.75 ± 11.47	85.70 ± 8.60	85.13 ± 5.69	76.63 ± 8.173
E/A	1.51 ± 0.29	0.68 ± 0.18**	1.24 ± 0.07^△△^	1.713 ± 0.37

Values are mean ± SD

LVPW; d: left ventricular end-systolic posterior wall thickness. LVPW; s: left ventricular end- diastolic posterior wall thickness. EF: ejection fraction. LVD; d: left ventricular end-diastolic diameter. LVD; s: left ventricular end-systolic diameter. E/A: ratio of mitral early inflow peak velocity to atrial contraction-related flow peak velocity

* *P* < 0.05 versus control, ** *P* < 0.01 versus control, ^Δ^, *P* < 0.05 versus Ang II, ^ΔΔ^, *P* < 0.01 versus Ang II

### Inhibitory effects of ANP on atrial fibrous tissue deposition and myofibroblast proliferation

**Fig. 2:**
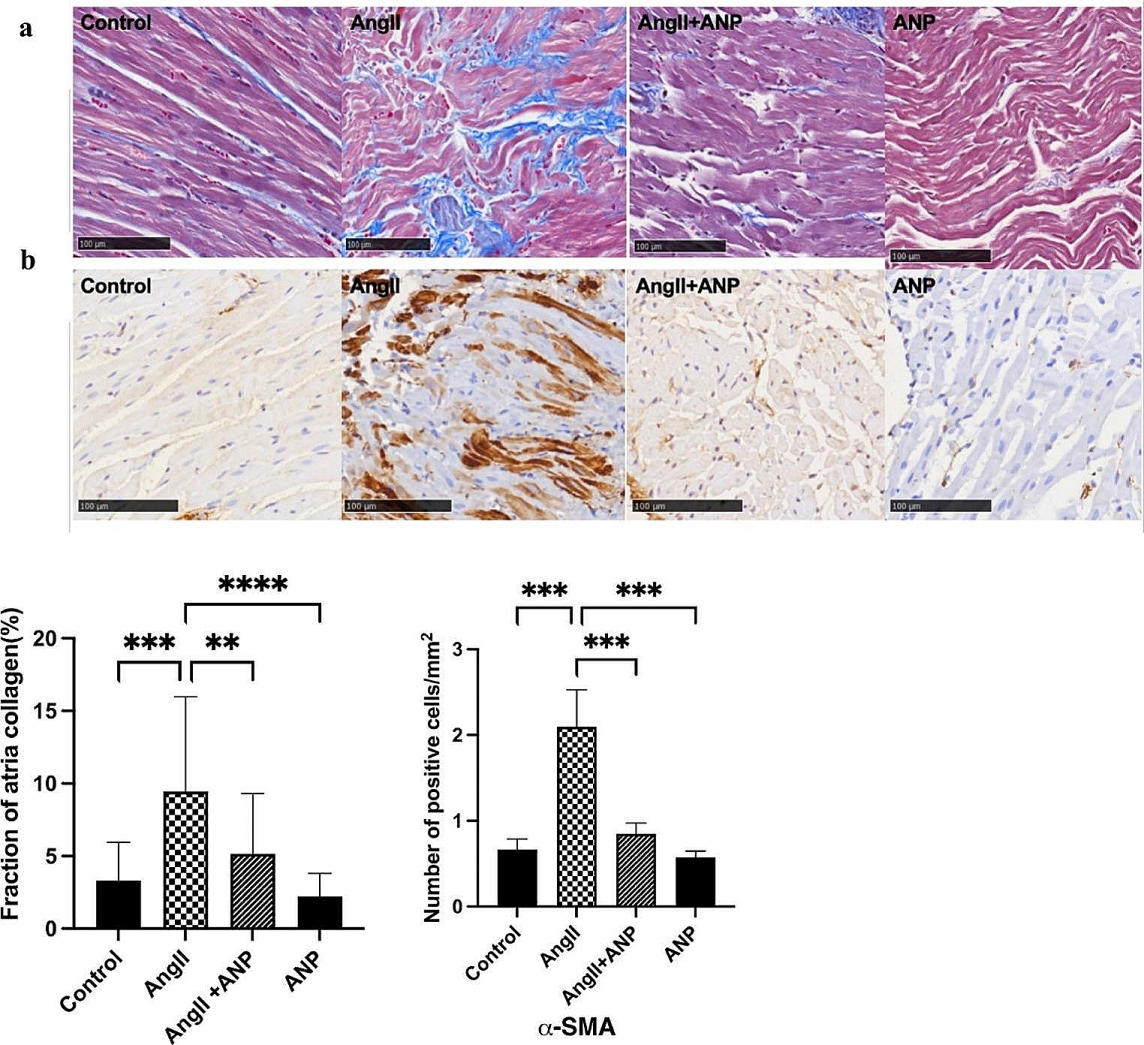
Histological features of Ang II-induced myocardial fibrosis. (**a**) Light micrograph of Masson and the percent area of myocardial interstitial and perivascular fibrosis. (**b**) Immunohistochemistry reveals alterations in the proliferation of atrial myofibroblasts. Control: control rats, Ang II: angiotensin II-treated rats, Ang II + ANP: Ang II plus atrial natriuretic peptide-treated rats, ANP: atrial natriuretic peptide-treated rats. * *P* < 0.05, ** *P* < 0.01, *** *P* < 0.001.

Histological assessments following Masson staining revealed significant findings in the deposition of extracellular matrix fibrous tissue in atrial tissues, identifiable by distinct blue staining. Compared to control rats, a marked increase in fibrous tissue deposition was noted in the atrial tissues of rats treated with Ang II, indicative of significant fibrosis. Importantly, co-infusion with ANP notably mitigated this collagen fiber content in atrial tissues, as observed in Fig. 2a.

Further analysis focused on the expression **of α-SMA **in myofibroblasts. Immunohistochemical labeling for α-SMA-positive cells showed a substantial increase in the number of positive cells in rats infused with Ang II alone. Remarkably, the concurrent administration of ANP led to a significant reduction in α-SMA-positive cells. However, there was no statistically significant difference in the number of positive atrial cells in rats receiving only ANP infusion compared to controls, as shown in Fig. 2b.

**Fig. 3:**
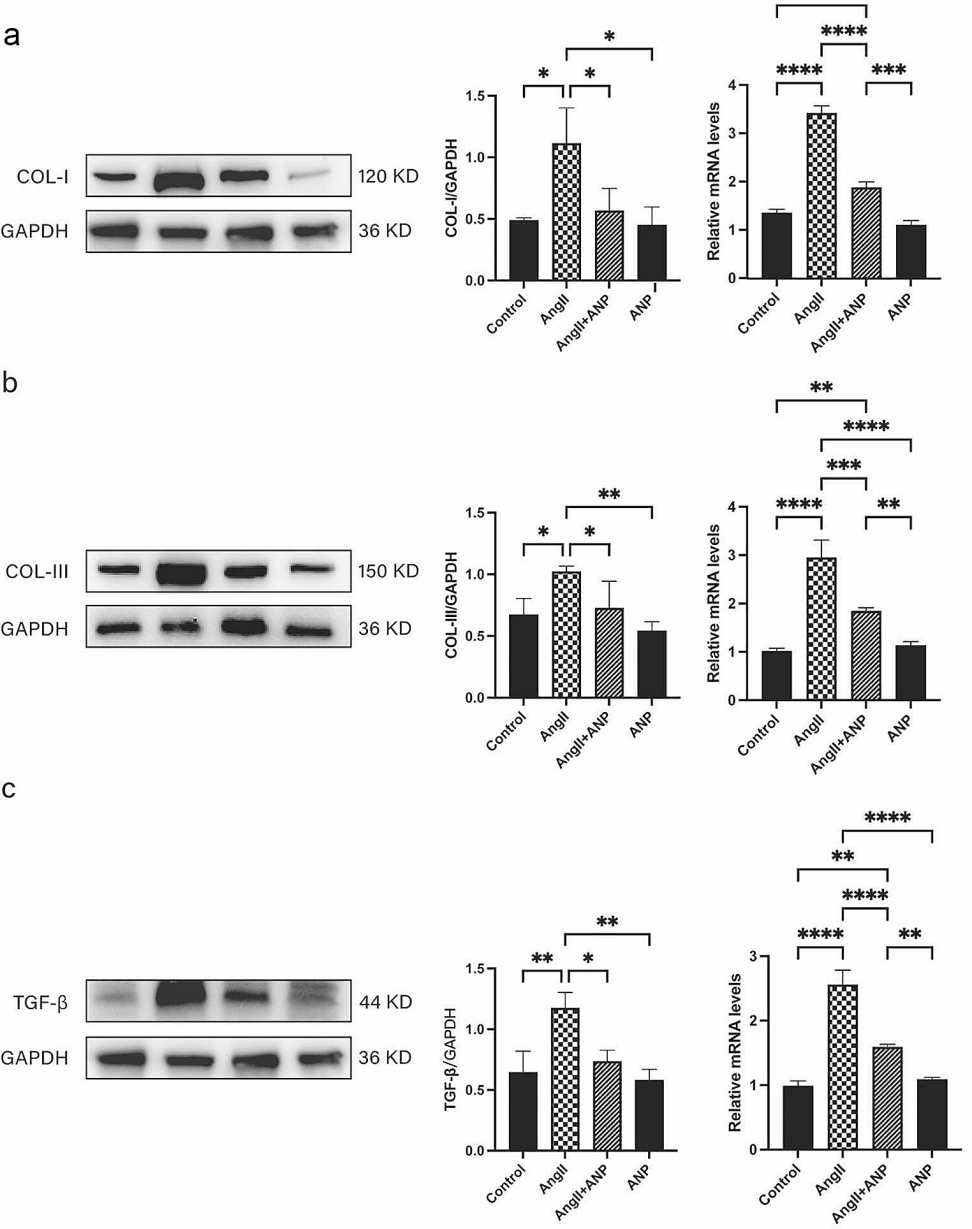
Western blot and qPCR demonstrate changes in atrial myocardial collagen proliferation in each group. (**a**) Changes in the expression of Collagen Type I. (**b**) Changes in the expression of Collagen Type III. (**c**) Changes in the expression of TGF-β. Control: control rats, Ang II: angiotensin II-treated rats, Ang II + ANP: Ang II plus atrial natriuretic peptide-treated rats, ANP: atrial natriuretic peptide-treated rats. * *P* < 0.05, ** *P* < 0.01, *** *P* < 0.001, **** *P* < 0.0001

Western blot and qPCR analyses provided additional validation. Compared to control rats, there was a significant upregulation in the expression of Type I, Type III collagen and TGF-β, both at the protein and mRNA levels, in the myocardial tissues of rats induced with Ang II. Treatment with ANP significantly alleviated these pathological changes, as depicted in Fig. 3.

### ANP’s role in reducing myocardial inflammation

**Fig. 4:**
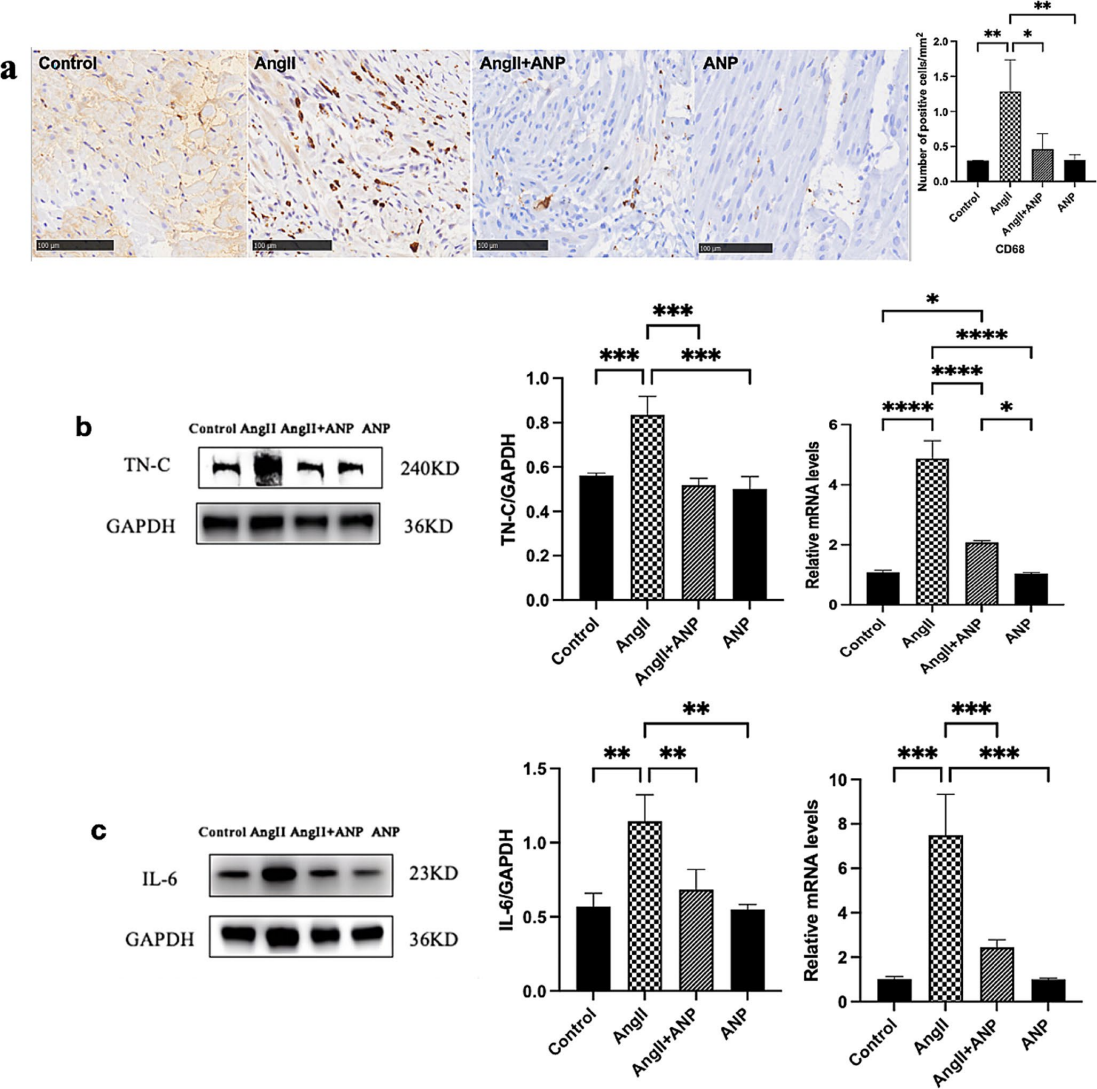
Inflammation changes in the atrium (**a**) IHC revealed changes in infiltration of macrophages in the atria (**b**) Western blot and qPCR demonstrate changes in the expression of TN-C. (**c**) Western blot and qPCR demonstrate changes in the expression of IL-6. Control: control rats, Ang II: angiotensin II-treated rats, Ang II + ANP: Ang II plus atrial natriuretic peptide-treated rats, ANP: atrial natriuretic peptide-treated rats. * *P* < 0.05, ** *P* < 0.01, *** *P* < 0.001, **** *P* < 0.0001

The impact of ANP on myocardial inflammation was assessed through several methodologies. Immunohistochemistry (IHC) was employed to evaluate macrophage infiltration in myocardial tissues. Compared to control rats, a significant increase in CD68-positive cells, indicative of macrophage infiltration, was observed in the atrial myocardium of rats treated with Ang II. Notably, ANP administration effectively attenuated this increased recruitment of myocardial macrophages, resulting in levels comparable to those in control rats, as illustrated in Fig. 4a.

Further, the expression levels of the inflammatory factors TN-C and IL-6 in atrial tissues were quantified using Western blot and qPCR analyses. These analyses revealed a pronounced increase in TN-C and IL-6 levels in the atrial tissue of Ang II-treated rats compared to controls. Following the co-administration of ANP, there was a noticeable decrease in the expression of both TN-C (Fig. 4b) and IL-6 (Fig. 4c), suggesting a significant reduction in the inflammatory response within the atrial tissue. Additionally, the study observed that the sole administration of ANP did not exhibit a discernible impact on the levels of TN-C and IL-6 in atrial tissues. This finding suggests that the anti-inflammatory effects of ANP are particularly pronounced in the context of Ang II-induced myocardial inflammation.

### Upregulation of the PI3K/Akt signaling pathway by Ang II

**Fig. 5:**
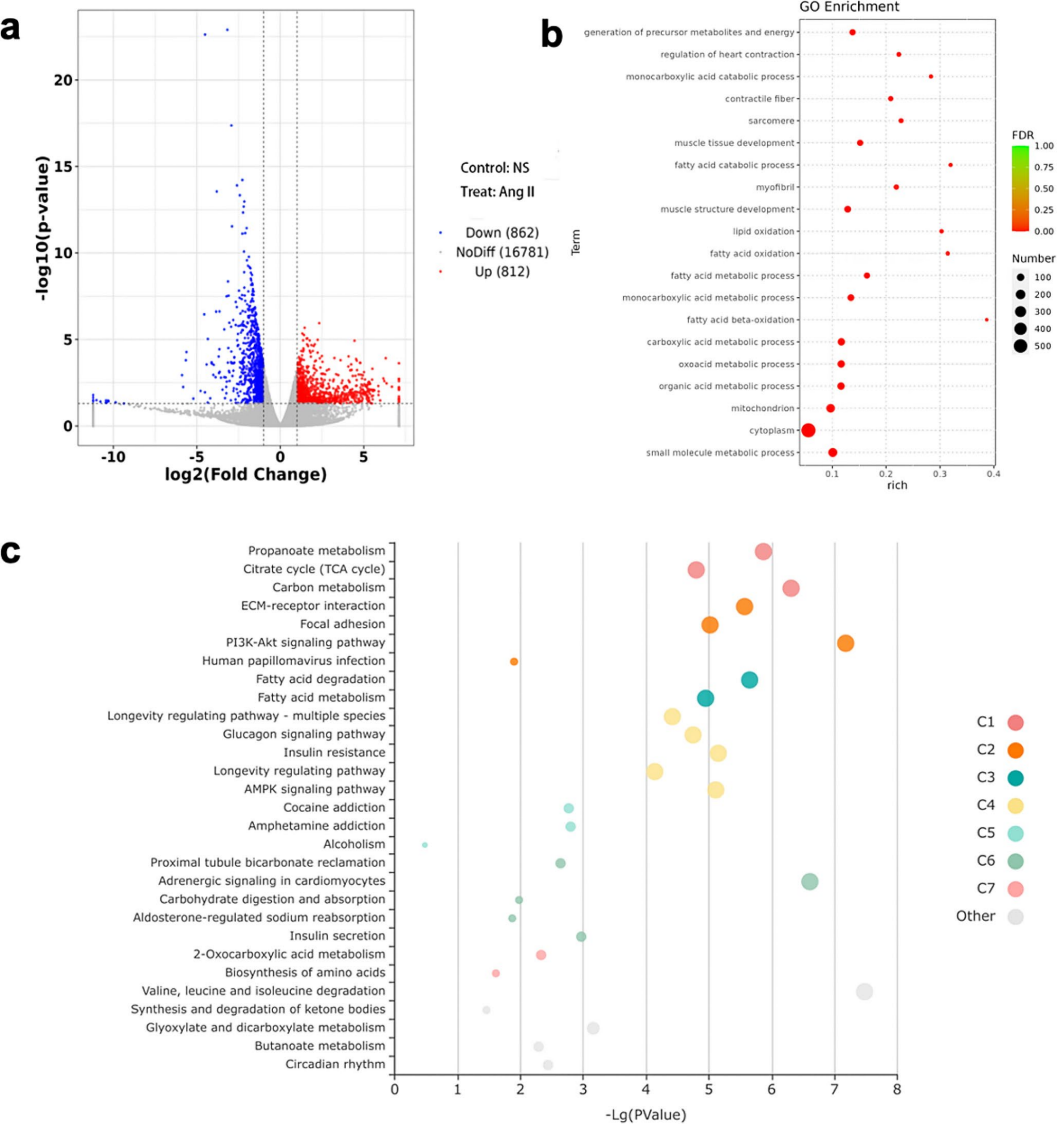
Eukaryotic transcriptome sequencing. (**a**) Gene expression differences between control and Ang II rats. (**b**) Functional enrichment analysis (GO Analysis) of differences between control and Ang rats. (**c**) Pathway enrichment analysis (KEGG Analysis) of differences between control and Ang II rats

Eukaryotic transcriptome sequencing analysis of atrial tissues from both control and Ang II-treated rats revealed significant changes in gene expression profiles. In the atrial myocardium of Ang II rats, there were 812 upregulated genes and 862 downregulated genes compared to controls. Notably, there was a marked upregulation in the expression of the TN-C gene (Fig. 5a). Functional enrichment analysis, specifically Gene Ontology (GO) analysis, highlighted significant alterations in genes related to myocardial fibrosis and regulation of cardiac contraction in the Ang II group (Fig. 5b). Additionally, pathway enrichment analysis using Kyoto Encyclopedia of Genes and Genomes (KEGG) indicated that the differences between the Ang II and control rats were predominantly associated with signaling pathways such as PI3K-Akt, AMPK, and related metabolic pathways (Fig. 5c).

### Inhibitory effects of ANP on the PI3K/Akt signaling pathway in cardiac fibroblasts

**Fig. 6:**
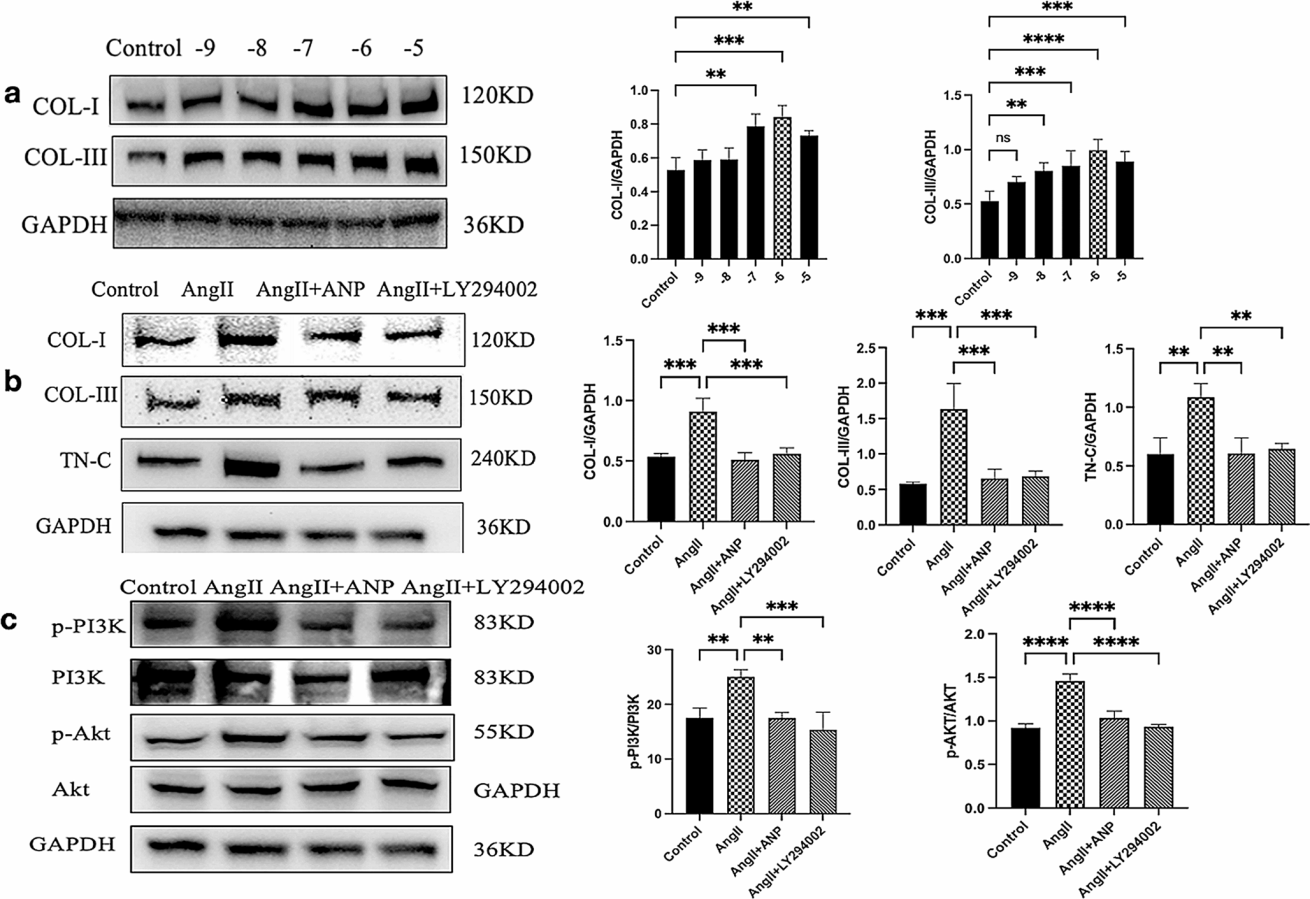
ANP and LY294002 inhibit Ang II-induced protein expression increase. (**a**) The effects of Ang II at different concentrations on collagen secretion. (**b**) ANP and LY294002 inhibit the secretion of collagen and TN-C. (**c**) ANP and LY294002 restored the phosphorylation of PI3K and Akt. Control: control fibroblast, Ang II: angiotensin II-treated fibroblast, Ang II + ANP: atrial natriuretic peptide-pretreated plus Ang II-treated fibroblast, Ang II + LY294004: LY294002-pretreated plus Ang II-treated fibroblast. * *P* < 0.05, ** *P* < 0.01, *** *P* < 0.001, **** *P* < 0.0001

The impact of ANP and the inhibition of the PI3K/Akt signaling pathway on collagen and TN-C secretion was assessed in Ang II-induced cardiac fibroblasts using Western blot analysis. Ang II administration led to a dose-dependent increase in the expression of Type I and Type III collagen, with the highest stimulation observed at an Ang II concentration of 10^-6 (Fig. 6a). To explore the role of the PI3K signaling pathway in inflammation, LY294002, a broad-spectrum PI3K inhibitor, was used to block this pathway. The results showed that both ANP and the blockade of the PI3K pathway inhibited the Ang II-induced secretion of collagen and TN-C (Fig. 6b). Furthermore, Ang II treatment increased the expression of phosphorylated PI3K and Akt, while ANP treatment reduced the phosphorylation of these signaling molecules (Fig. 6c).

## Discussion

Our study highlights the potent inhibitory effects of ANP on myocardial inflammation, particularly through the suppression of inflammatory factors like TN-C and IL-6, and the reduction of macrophage infiltration. This inhibition counters the myocardial inflammation induced by Ang II, suppresses the proliferation of Type I and Type III collagens in atrial tissue, reduces the activation of the TGF-β signaling pathway and diminishes the activation of cardiac fibroblasts. Consequently, ANP emerges as a pivotal agent in anti-inflammatory responses, effectively reversing atrial fibrosis. Moreover, our findings suggest that ANP also has protective effects on diastolic cardiac function, representing a promising therapeutic avenue for atrial fibrosis.

The involvement of myocardial fibrosis in the pathophysiological mechanisms of various cardiovascular diseases, especially atrial fibrosis, is widely recognized. Atrial fibrosis disrupts the continuity of atrial muscle fibers, leading to local conduction abnormalities, increased atrial electrical heterogeneity. Furthermore, excessive collagen accumulation in atrial fibrosis diminishes tissue elasticity, stiffening the atrial wall. This stiffening of the atrial wall can impair both contraction and relaxation, potentially affecting atrial filling and increasing atrial pressure and the potential development of atrial arrhythmias [22]. The pathogenesis of atrial fibrosis is driven by a multitude of complex mechanisms including inflammation, oxidative stress, and injury repair processes [23]. The role of inflammation in the development of atrial fibrosis has garnered significant attention in recent years. Various cytokines associated with chronic inflammation are known to contribute to the formation of atrial fibrosis. IL-6 infusion in rat models has been linked to increased myocardial wall thickness, deepened fibrosis, and significant impacts on myocardial remodeling [24–26]. Furthermore, macrophages play a crucial regulatory role in myocardial inflammation and have been implicated in the progression of myocardial fibrosis [27]. For instance, inhibiting the NLRP3 inflammasome activation in macrophages has been shown to prevent the transformation of fibroblasts into myofibroblasts [28], and specific deletion of Doublecortin-like kinase 1 (DCLK1) in macrophages has demonstrated a reduction in cardiac hypertrophy and myocardial fibrosis [29].TN-C, a profibrotic and proinflammatory modulator, not only weakens myocardial cell adhesion to connective tissues but also plays a crucial role in myofibroblast recruitment and aggregation [30–32]. It amplifies Ang II-induced cardiac inflammation, promoting the production of pro-inflammatory factors such as IL-6 and macrophages, thus influencing macrophage polarization and intensifying macrophage-mediated myocardial fibrosis [32,33]. Studies have also shown that inhibiting TN-C expression can mitigate ventricular fibrosis following myocardial infarction, reduce liver fibrosis induced by hepatitis, and alleviate joint fibrosis in rheumatoid arthritis [33–36].

Inflammatory factors are direct inducers of atrial fibrosis, suggesting that inhibiting myocardial inflammation could be a viable strategy to reverse atrial fibrosis. The anti-inflammatory effect of ANP has been observed in various animal models, showing alleviation of inflammation in tissues like the pancreas, colon, and myocardium [37–39]. Clinical studies have indicated that ANP infusion can mitigate spontaneous inflammation following myocardial infarction, reducing ventricular fibrosis and counteracting adverse ventricular remodeling [19]. Therefore, ANP emerges as a potential therapeutic measure against inflammation and fibrosis. While limited research exists on ANP’s impact on atrial fibrosis specifically, our study reveals that Ang II induces atrial inflammation, increases inflammatory factor expression, and enhances atrial fibrosis. The administration of ANP significantly alleviates myocardial inflammation and reverses the degree of atrial fibrosis. Our results suggest a therapeutic effect of ANP in mitigating inflammation and atrial fibrosis.

A study has shown that the levels of TN-C in the left and right atria of patients with AF are significantly higher than those in the serum and are markedly elevated compared to the femoral artery and vein, indicating a certain specificity of TN-C in the atria [40]. Given that TN-C not only directly induces fibrosis but also enhances the fibrogenic capability of other inflammatory factors, we propose that ANP may exert an anti-inflammatory effect by downregulating TN-C expression, thereby suppressing atrial fibrosis. To further validate the impact of ANP on TN-C and fibrosis, we utilized in vitro cultured cardiac fibroblasts. We found that ANP pretreatment reversed the Ang II-induced upregulation of TN-C, Type I collagen, and Type III collagen in fibroblasts. Thus, ANP inhibits the secretion of TN-C induced by Ang II, alleviating inflammation, and suppressing the fibrotic process. However, it is essential to recognize the complex regulatory mechanisms governing inflammation and fibrosis, involving numerous signaling molecules and pathways. Based on transcriptome sequencing results from our study, we observed a significant increase in the expression of the PI3K/Akt signaling pathway following Ang II infusion. We hypothesize that ANP may exert its anti-inflammatory and anti-fibrotic effects by inhibiting the Ang II-activated PI3K/Akt-TN-C pathway. Our experiments using cardiac fibroblasts revealed that Ang II increased the expression of p-PI3K, p-Akt, and TN-C protein, activating fibroblast fibrosis. Significantly, pretreatment with ANP restored the phosphorylation of PI3K and Akt. These observations emphasize the potential of ANP in regulating fibrosis induced by Ang II through the PI3K-Akt-TN-C signaling pathway. The PI3K/Akt signaling pathway is involved in various inflammatory processes and is associated with multi-organ fibrosis. Current research indicates that blocking this pathway can alleviate fibrosis in the liver, lungs, and renal interstitium [41–43]. Our results showed that TN-C and collagen secretion induced by Ang II were suppressed when fibroblasts were pretreated with LY294002, which indicates that blocking the PI3K signaling pathway suppresses Ang II-induced inflammation and fibrosis. These findings collectively suggest the involvement of the PI3K/Akt-TN-C signaling pathway in ANP-mediated regulation of fibrosis, offering new insights into potential therapeutic strategies for managing atrial fibrosis.

Additionally, in the conducted experiments, the impact of ANP on Ang II-induced hypertension was assessed. It was observed that ANP significantly inhibited atrial fibrosis without markedly lowering blood pressure, suggesting its anti-fibrotic actions may primarily occur through non-hypotensive pathways. This insight underscores the potential of ANP in treating atrial fibrosis independently of blood pressure modulation, advocating for future studies on its direct effects on cardiac cells and molecular signaling. And no significant differences in EF were noted across experimental groups, hinting that the short, two-week Ang II exposure might not suffice for substantial ventricular structural changes, highlighting the necessity for longer-term assessments to observe significant alterations in ventricular structure and function, which was consistent with the findings of previous studies [19].

## Conclusion

This study elucidates the therapeutic potential of ANP in mitigating myocardial inflammation and ameliorating atrial fibrosis. The mechanism of action of ANP in combating atrial fibrosis involves, at least partially, the suppression of the Ang II-induced PI3K/Akt-TN-C signaling pathway, a key mediator of myocardial inflammation. Notably, ANP’s administration did not result in significant hypotensive effects, highlighting its favorable safety profile. Consequently, ANP stands out as a novel, effective, and safe adjunctive therapy, promising significant advancements in the clinical management of atrial fibrosis. This approach holds the potential to fundamentally reduce the incidence and recurrence of atrial fibrillation.

### Innovation

This study systematically explores treatment strategies for atrial fibrosis, emphasizing the role of ANP in anti-inflammatory action and the reversal of atrial fibrosis. Through transcriptomic sequencing and cellular experimentation, we have uncovered that the activation of the PI3K/Akt-TN-C signaling pathway constitutes one of the pivotal mechanisms underlying cardiac inflammation and fibrosis during the process of atrial fibrosis. ANP was found to inhibit this pathway’s activation, thereby attenuating the severity of cardiac inflammation and fibrosis. These discoveries provide novel targets and avenues for therapeutic intervention in future research. Moreover, the administration of ANP did not significantly alter blood pressure, indicating its potential clinical applicability and its promise as a significant therapeutic and management intervention to ameliorate atrial fibrosis.

### Limitations

However, there are certain limitations to our study that warrant consideration. The inhibition of the PI3K signaling pathway by ANP was validated only in cellular experiments and was not further examined in animal models. And due to experimental constraints and resource limitations, our study did not directly assess the inducibility of atrial fibrillation or changes in sinoatrial node function. We did not explore the morphological changes of myofibroblasts following Ang II treatment, which could complement the biochemical and molecular findings. Furthermore, our study primarily focused on atrial changes, and therefore, we did not directly measure levels of ventricular fibrosis and the blood level of inflammatory factors and profibrotic mediators. While animal and cell models offer valuable insights, they may not entirely replicate human physiological conditions, potentially leading to variances in the applicability of our findings to human patients. Therefore, further research, including clinical trials, is essential to fully ascertain the efficacy of ANP as a therapeutic agent for atrial fibrosis.

## Electronic supplementary material

Below is the link to the electronic supplementary material.

Supplementary Material 1

## Data Availability

No datasets were generated or analysed during the current study.
