# Supplementary material for: Targeting myocardial inflammation: investigating the therapeutic potential of atrial natriuretic peptide in atrial fibrosis
Source: Mol Biol Rep. 2024 Apr 15;51(1):506. doi: 10.1007/s11033-024-09393-w (PMC11018689; doi:10.1007/s11033-024-09393-w)

**Figure 1**.The original western blot for COL-I and COL-III in Fig.3


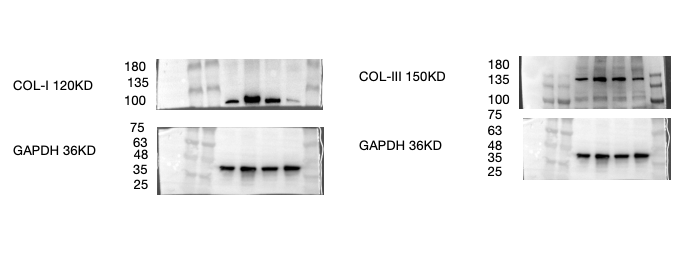


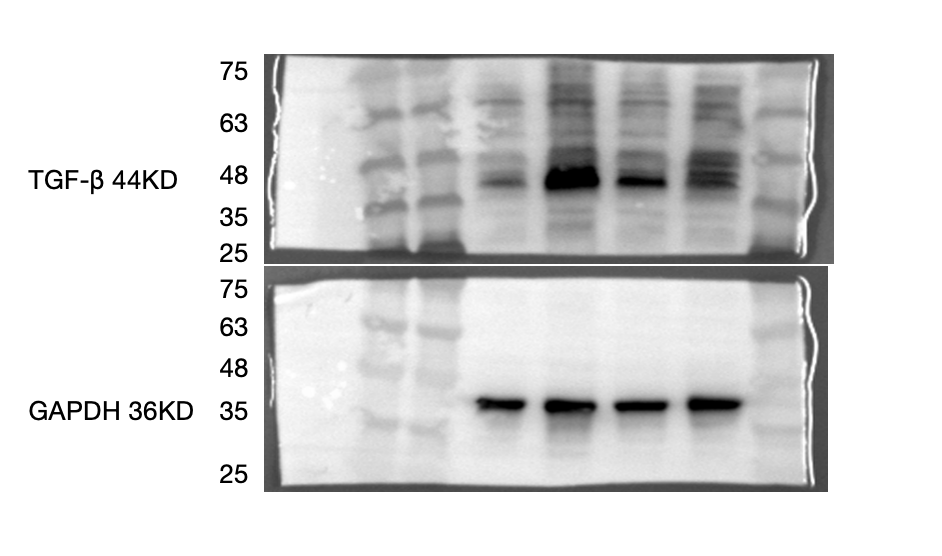


**Figure 2**.The original western blot for TN-C and IL-6 in Fig.4


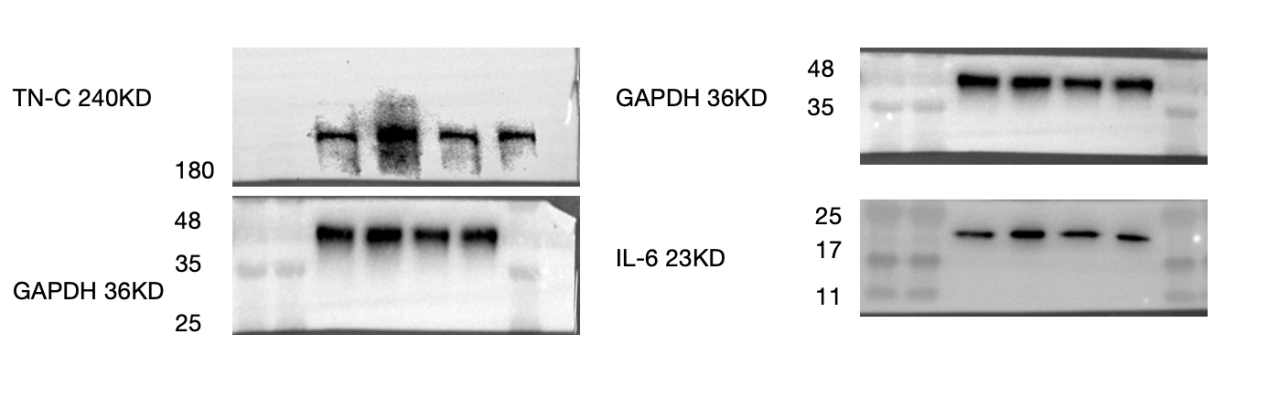


**Figure 3**.The original western blot for COL-I， COL-III，TN-C，p-PI3K/PI3K and p-Akt/Akt in Fig.6


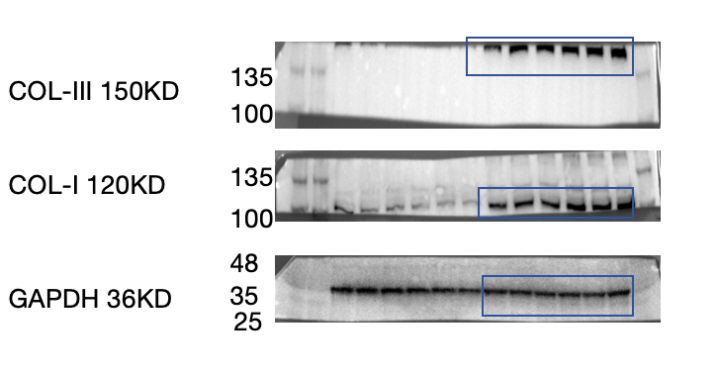

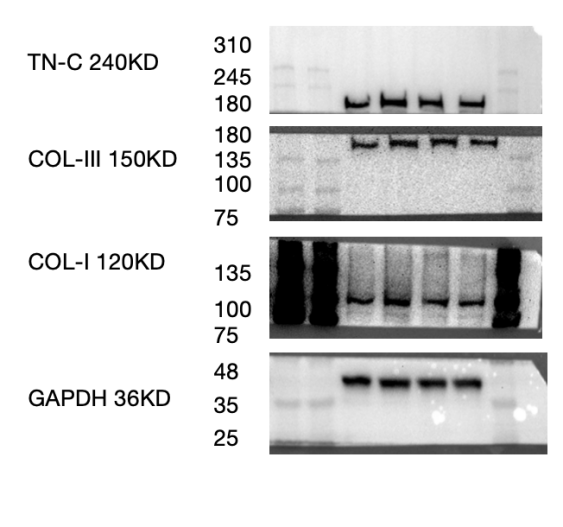


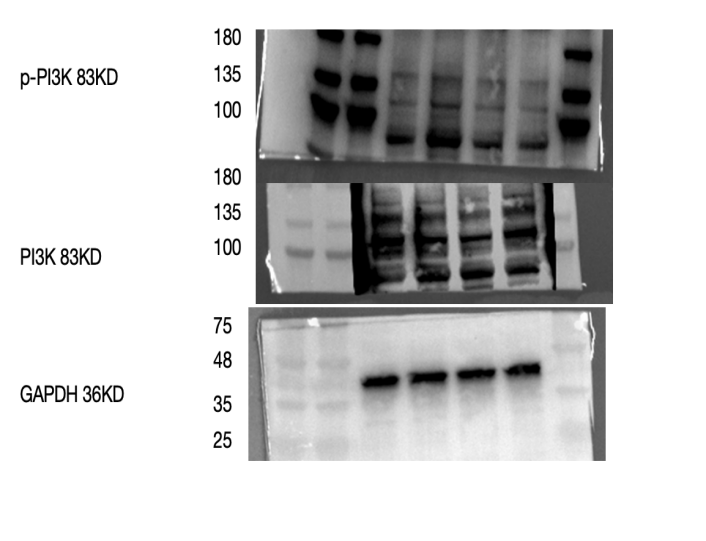

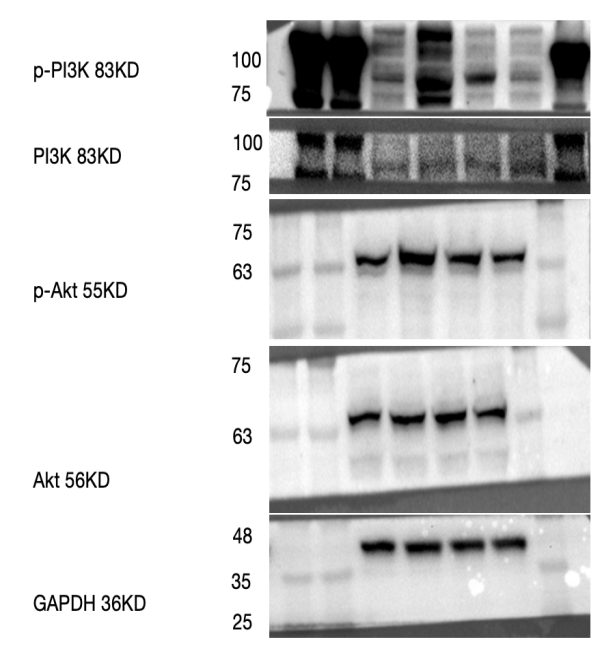

Supplement: Supplementary file 1 — Supplementary Material 1 [file 11033_2024_9393_MOESM1_ESM.docx]
